# Quantifying the Impact of Spatial Disorientation on Pilot Mental Workload and Attentional Focus

**DOI:** 10.1177/00187208251323116

**Published:** 2025-04-10

**Authors:** Fleur W. Evertsen, Annemarie Landman, Eric L. Groen, Mark M. J. Houben, M. M. (René) van Paassen, Olaf Stroosma, Max Mulder

**Affiliations:** 1 2860 Delft University of Technology, The Netherlands; 2 312992 TNO, The Netherlands; 3 Cranfield University, UK

**Keywords:** situational awareness, vertigo, aviation, distraction, aerospace, aircrew

## Abstract

**Objective:**

We aimed to find objective measures of the impact of spatially disorienting (SD) stimuli on pilot cognition in an ecologically valid environment.

**Background:**

SD frequently occurs in military rotary-wing operations and often contributes to mishaps. Effects of SD stimuli on pilots are usually quantified using control errors, but effects on cognition have not yet been successfully quantified.

**Method:**

Military helicopter pilots (*n* = 14) performed scenarios with six SD stimuli (SD condition) and six corresponding control stimuli (NoSD condition) in a motion-base simulator with integrated virtual reality headset. SD stimuli were: false horizon, featureless terrain, leans, brownout, a somatogyral yaw illusion, and loss of horizon due to night vision goggles (NVGs). Mental workload was measured using auditory arithmetic task performance and attentional focus was measured using eye-tracking.

**Results:**

Average arithmetic task performance was significantly impaired, and proportional gaze dwell time on the attitude indicator was significantly increased in the SD compared to the NoSD condition. Of the six SD stimuli, the featureless terrain, the leans, and the brownout induced significant effects on performance, whereas the featureless terrain, brownout, and false horizon significantly affected gaze behavior. The NVGs and somatogyral yaw stimuli did not induce significant effects. Pilots’ self-reports indicated awareness of all SD stimuli, except for the featureless terrain.

**Conclusion:**

The results indicate that SD impacts pilot mental workload and attentional focus.

**Application:**

Modern military aircraft present a large volume of mission-related information to pilots. This study shows that SD stimuli may negatively impact the processing of such information.

## Introduction

Spatial disorientation (SD) in aviation refers to the incorrect perception by the pilot of the aircraft orientation or motion relative to the Earth’s surface (Previc & Ercoline, 2004) or, when flying in formation, relative to another aircraft (Benson, 1988). SD occurs due to sensory illusions caused by limitations of the human sensory system. In the most extreme cases it can result in fatal accidents, either through loss of control in-flight (LOC-I) or controlled flight into terrain (CFIT). SD is a major contributor to incidents and accidents within general, civil (Belcastro et al., 2017) and military aviation (Poisson & Miller, 2014). Incidents per flight hour are typically higher in military aviation, as this is characterized by higher workload and more aggressive maneuvers. Military rotary-wing operations are especially susceptible to SD, with a 5.73 times higher incidence rate than fixed-wing (Poisson & Miller, 2014). Bushby et al. (2018) reported 43% of rotary-wing aircraft accidents in the UK Military between 2000 and 2015 to involve SD.

**Figure 1.**
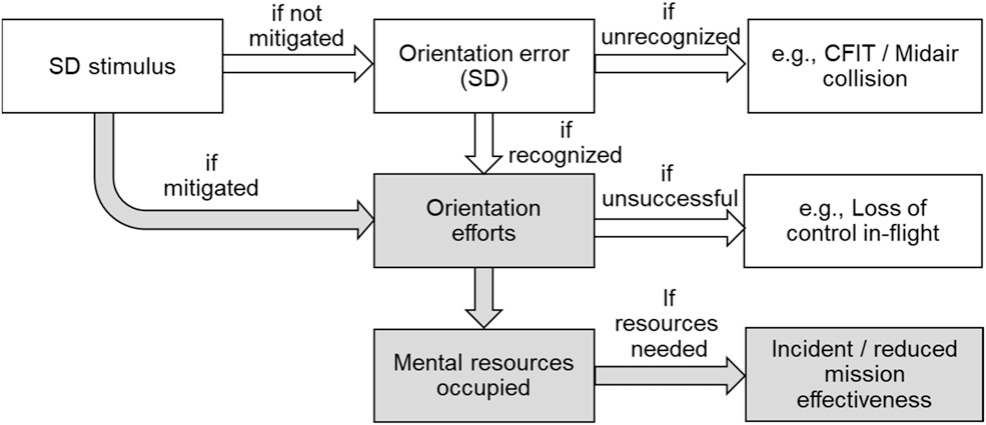
Schema representing the hypothesized effects of SD on pilot performance. The white elements represent the effect of SD on control errors, potentially leading to controlled flight into terrain (CFIT), midair collisions, or loss of control in-flight. The gray elements represent the effects of SD stimuli on the pilot’s mental resources, potentially leading to incidents or decreased mission effectiveness. These grey elements are the topic of the current study.

When quantifying the effects of SD on pilots, many studies have focused on the effects of SD stimuli on orientation errors and subsequent inappropriate control inputs (see, e.g., Boril et al., 2020; Landman et al., 2019; Lewkowicz et al., 2020; Figure 1, white elements). Orientation errors can remain unrecognized (Type-I SD) or recognized (Type-II SD) after which orientation efforts may either succeed or fail (Type-III SD; Previc & Ercoline, 2004). The current study focuses on a different pathway through which SD stimuli may impair pilot performance (see Figure 1, grey elements). Here, the pilot’s orientation efforts to mitigate the effect of an SD stimulus occupy cognitive resources (e.g., attentional focus and working memory) which cannot be used for other tasks. This assumption is based on the “orientation first” principle, which states that attention will involuntarily be directed towards maintaining spatial orientation when this is being disturbed (Gresty et al., 2003). This means that, even when SD does not lead to control errors, it can draw the pilot’s attention away from other tasks, such as navigation, communication and, in military aircraft, tactical operations. The latter is particularly relevant because modern military aircraft are equipped with advanced sensor technologies and datalink capabilities, presenting a wealth of tactical information to the pilots. When their information processing capacity is being impaired by SD, this may impact the mission effectiveness.

Previous laboratory studies showed that mental workload increased during the presentation of vestibular motion stimuli (Gresty et al., 2008), presented in darkness (van Elk & Blanke, 2014; Yardley et al., 2002), or when presenting rotating or translating visual fields (Ehrenfried et al., 2003; Gresty et al., 2008; Yardley et al., 1999, 2002). However, these studies did not explicitly test the effect of orientation errors. This was done in a study using a rotating chair by Landman et al. (2024), who found that SD led to a 31% decrease of arithmetic performance speed with a large effect size. The “orientation first” principle was also tested in flight simulator studies. Webb et al. (2012) compared formation flights with severe maneuvering against formation flights with less severe maneuvering, both with covered cockpit instruments. They found an effect on secondary task performance, but did not control for complexity of the flying task. Strozak et al. (2018) tested the effect of six SD stimuli presented to military pilots in a motion-based simulator. SD significantly decreased performance on secondary tasks (i.e., tone duration discrimination task and N-back task) during one of the stimuli (i.e., the leans), and there were trends towards this effect during a somatogyral and a false horizon stimulus. Recently, Fudali-Czyż et al. (2024) tested whether the same six SD stimuli impacted performance on a visual change detection task in military pilots, and found significant effects on attention distribution but not on mental workload.

Besides secondary task performance, pilot gaze behavior could also be useful as an objective measure of the impact of SD on mental workload, as it could indicate whether attention is focused on information of spatial orientation. Bałaj et al. (2019) found differences in gaze distribution on the flight instruments in three out of six analyzed profiles (i.e., false horizon, somatogyral yaw, and Coriolis illusion) but there was no conclusive pattern in the direction of this distribution (e.g., towards attitude information during SD). In an exploratory study with six military student pilots, Ledegang and Groen (2018) found indications of increased gaze directed towards the attitude indicator for two of five stimuli (i.e., somatogravic and false horizon).

Concluding, effects of SD on mental workload have been demonstrated in laboratory studies, but simulator studies have either been exploratory or inconclusive. The main objective of this study was therefore to build upon these previous studies by quantifying effects of different types of SD stimuli on pilot mental workload and attentional focus in a simulator environment. A secondary task paradigm was used to measure mental workload (Williges & Wierwille, 1979), with flying or monitoring being the primary task. The secondary task was an auditory mental arithmetic task. This is a mental tracking-type task, requiring both the storing of information in working memory and processing of this information (Lezak et al., 2004). It was selected to represent piloting tasks of processing aurally communicated information, while also yielding consistent performance data in a controlled manner. We used gaze behavior as an indicator of attentional focus and tested whether pilots looked more towards the attitude indicator during SD stimuli.

Our secondary objective was to explore the effectiveness of six SD stimuli, of which three were based on Strozak et al. (2018). In contrast to their study, no covering of the displays was used to disorient pilots, and we aimed to measure the effect of SD on cognitive task performance more precisely by specifically targeting the 30s time frame during which each SD stimulus was presented.

## Method

### Participants

The sample group consisted of 14 helicopter pilots (all male, seven captains and seven copilots; see, Table 1) who each possessed a military pilot license. A power analysis for a paired-samples *t* test and a large effect size (as based on Landman et al., 2024) indicated that a sample group of around 15 participants would be sufficient. Nine flew the AS532-U2 Cougar, two the CH-47 Chinook (both types Royal Netherlands Air Force), and two flew the NH90 (Royal Netherlands Navy). None of these pilots had experience with flying the simulated helicopter type used in this study. All but four participants had experience with flying in VR. Six participants had worked as flight instructors. This study complied with the tenets of the Declaration of Helsinki and was approved by the Ethical Review Board of TNO Soesterberg under number 2023-059.

**Table 1.** Sample Group Characteristics.

	Mean	SDev
Age	34.4	7.0
Flight hours	1513	1068
Years of experience	10.0	7.2

SDev = standard deviation.

### Apparatus

**Figure 2.**
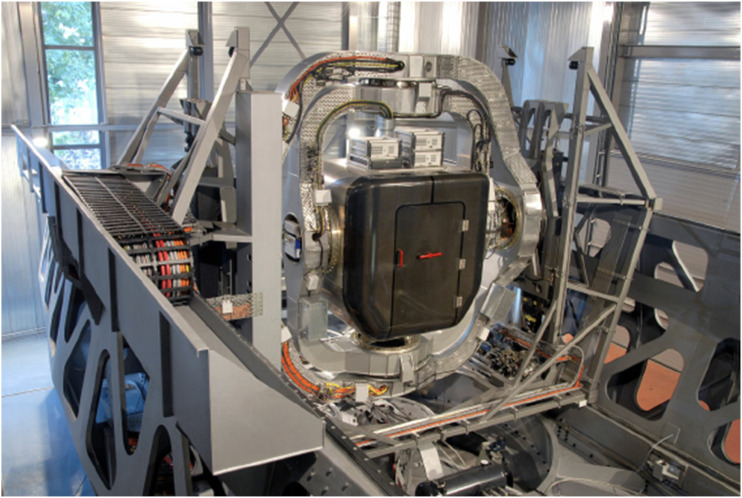
The Desdemona motion simulator.

The experiment was performed using the Desdemona motion simulator, constructed by AMST-Systemtechnik GmbH (Ranshofen, Austria) and located in Soesterberg, the Netherlands (Figure 2). Desdemona is a moving base simulator and has unique motion features due to its capabilities to combine the centrifuge design for sustained G-loading with five additional degrees-of-freedom. All motion features were used in this study; however, the centrifuge was not applied to generate g-forces. The cabin of Desdemona featured the AH-64 Apache collective and cyclic, and standard pedals. An AH-64 flight model (multiSIM B.V., Soesterberg) was used. It presents a high-fidelity simulation of the Delta and Echo version of the AH-64 and is used by the Royal Netherlands Air force for training.

**Figure 3.**
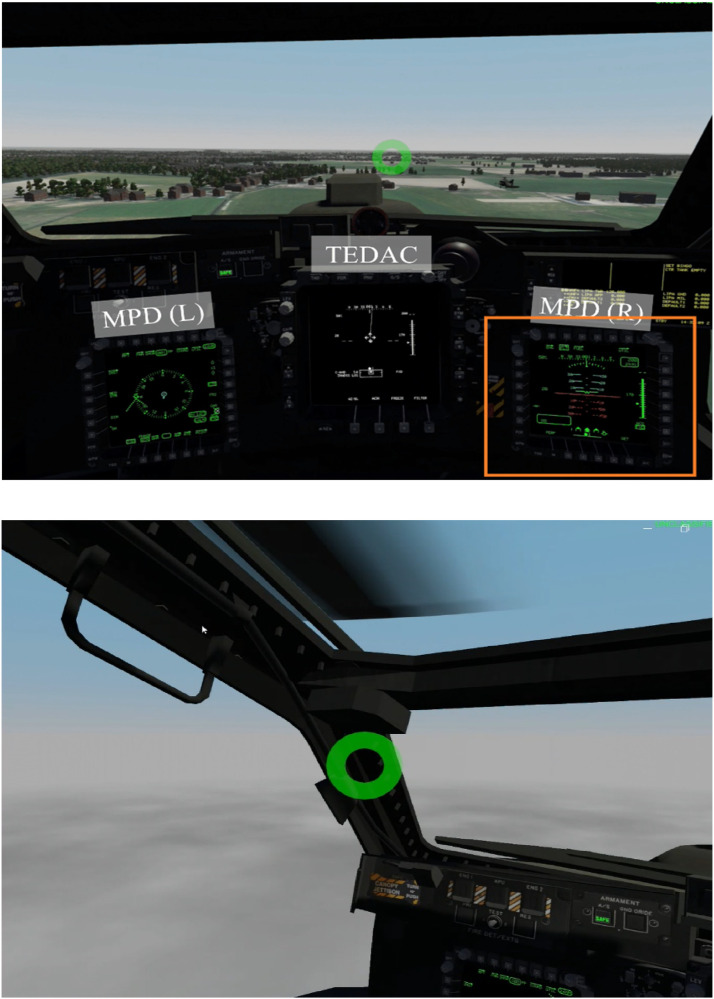
Top: the virtual front window and front seater cockpit of the AH-64 with the area of interest (orange square) around the flight page (including the attitude indicator) defined for gaze analysis. Bottom: the left side window. The green circle is not visible to participants, it indicates the gaze direction.

Visuals were presented in VR using the Varjo Aero (Varjo, Helsinki, Finland) VR headset, which has a 115° field of view (Figure 3). The rotation of the headset, as reported by its inertial measurement unit, was compensated by inverting the simulator angles, which are known within the motion queing. Yaw drift was compensated using two passive markers attached to the headset, while pitch and roll were corrected based on the gravity vector. Translation of the headset was not tracked. To ensure simulation of natural viewpoint movement, headset rotations were applied to the base of a rod, which simulates the offset between the neck’s rotational center and the eyes of the participant.

The built-in eye tracker was used to measure participant’s gaze with a sample rate of 200 Hz. The multi-purpose displays (MPDs) and the “Target Acquisition and Designation Sights” Electronic Display and Control (TEDAC) in the virtual world are highlighted in Figure 3. In this experiment, the Tactical Situation Display (TSD) was presented on the left MPD and the flight page on the right MPD. The TEDAC presented flight information but no sensor images. The flight page shows the attitude indicator, as well as aircraft altitude, speed, and heading. No helmet-mounted display (HMD) was used.

### Experimental Design

A within-subject design was used. All participants performed a cognitive task (see the Auditory Cognitive Task section) during flight scenarios that were performed once in an SD and once in an NoSD condition (see the Procedure section). Each scenario in the SD condition featured one of six SD stimuli (see the Stimuli section), whereas those in the NoSD condition featured corresponding NoSD stimuli. Auditory cognitive task performance, gaze behavior, control inputs, and subjective measures (see the Dependent Measures section) were compared between the SD and NoSD conditions. Exploratively, SD-NoSD comparisons were performed for each stimulus separately.

### Procedure

For each pilot the experiment lasted 3 hours, including briefing, familiarization, a test session with a break, and debriefing.

#### Briefing

In a 20-min briefing, the participants were informed that the goal of the experiment was to investigate effects of different flight maneuvers on their cognitive performance. The instructed flying task was to follow a designated fight path. The AH-64 flight controls and systems were outlined and the general display configuration was demonstrated.

#### Familiarization

Participants were placed in the virtual copilot/gunner position (frontseater) of the AH-64, since that best resembles the outside view of the NH90, AS532-U2, and CH-47. Virtual seat height was adjusted so that all participants had the same viewpoint. The familiarization session consisted of flying two circuits. In between the circuits the cognitive task was practiced 2 times, with the helicopter on the ground. The second circuit was flown with lower visibility. The cognitive task was practiced twice in-flight during the downwind stretch of the circuit.

#### Test Session

**Figure 4.**
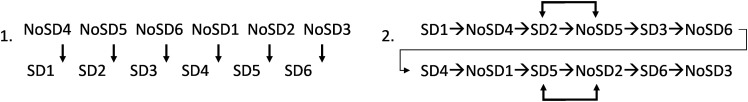
The sequencing of the SD and NoSD runs in half of a simulator session (e.g., before the break), with “1–6” being the six stimuli. After step 1 (left) four runs are switched (right) to prevent SD-NoSD conditions alternating in a predictable manner.

The test session consisted of 24 180-s experimental runs, that is, six SD stimuli and six NoSD stimuli, all performed twice to stabilize cognitive task performance results. The SD or NoSD stimulus was always presented at *t* = 90–120 s. Participants performed the cognitive task at *t* = 30–60 s, *t* = 90–120 s and *t* = 150–180 s. Each run was preceded by brief instructions on the required flight path. The six SD stimuli were balanced using a Latin Square design. The NoSD stimuli were then intermixed in this design, offset by four runs from their SD counterpart (Figure 4(a)). To limit predictability due to conditions alternating, the third and fourth run, as well as the ninth and tenth run were then switched around (Figure 4(b)). Every stimulus was featured once before and once after the break, with a different order. Every order of runs was used for a second participant, but with the conditions (SD/NoSD) switched. At the end, the actual goal of the experiment was explained and participants filled in a questionnaire.

### Stimuli

The six SD stimuli included: False horizon; the leans; somatogyral effect; reduced visibility due to night vision goggles (NVGs): brownout condition; and featureless terrain. All stimuli, except the somatogyral stimulus, have been frequently reported by military pilots (Pennings et al., 2020). Three SD stimuli (i.e., false horizon, leans, and somatogyral) have shown promising effects in a simulator study by Strozak et al. (2018).

#### False Horizon

The task was to fly straight and level above a cloud layer, heading 310°, speed 95 kts at 4800 ft altitude, and to scan outside for traffic. Traffic (a AS532-U2 Cougar) would be visible at *t* = 60 s to instill alertness. Only in the SD condition, the cloud layer became sloped so that its attitude at *t* = 90 s was 6° in roll and 3° in pitch. Pitch was added to blend the cloud layer with the surrounding cloud deck. After *t* = 120 s, the cloud layer attitude returned to level. The gradual fading in and out of the sloped cloud deck made it difficult to perceive these transitions.

#### Featureless Terrain

Participants were tasked with flying at a heading of 200°, a speed of 95 kts at an altitude of 500 ft over the sea, with a coast line visible ahead. At *t* = 60 s, the participant was instructed to turn right, heading 290° with 30° angle of bank. This turn ended with the helicopter facing the ocean with no land visible at *t* = 90 s. In the SD condition, the water did not contain reflection, texture and flow, to simulate a flat ocean. Such featureless terrain induces misperception of the altitude and possibly pitch angle (see, e.g., Gibb, 2007). After *t* = 120 s, the participant was instructed to turn left, heading 200° with 30° angle of bank, so that the coastline was visible again.

#### The Leans

The task was to fly straight and level, heading 080°, speed 115 kts, and at 4000 ft altitude. The helicopter was in the clouds, simulated by solid grey visuals. Only in the SD condition, the simulator cabin was tilted right with a rate of 1.5°/s so that the roll angle was 15° at *t* = 90 s. The onset and continuation of the roll motion was perceptible. The tilt of the cabin caused a mismatch between vestibular cues and instrument information. After *t* = 120 s, the simulator cabin was returned to level again with the same rate.

#### Brownout

Participants were tasked to monitor instead of actively fly, as looking outside during manual control in a brownout would not be according to procedures. The helicopter was following a leading helicopter at a distance of 150 m three times into a low-altitude hover at approximately 30 ft altitude. During the second low-altitude hover at *t* = 90–120 s, dust would spray up. Only in the SD condition, dust particle contrast was increased and tailwind was added to induce vection when looking outside, and dust opacity was increased so that the horizon was no longer visible. To ensure that the participant would perceive this stimulus, the task was given to track the clock (positioned immediately next to the window) and indicate a “go” at *t* = 125 s.

#### Somatogyral Yaw Effect

To apply the helicopter motions without the participant being aware, the participant had a monitoring role instead of a flying role. The scenario started in the clouds at 6200 ft altitude. The MPDs were set to present the performance page and engine page so that no information on flight path was shown. In the SD condition, the simulator was accelerated around the yaw axis for 75 s at a sub-threshold rate of 0.33°/s^2^ with an arm of 8 m and the front of the cabin directed towards the center. At *t* = 90 s, this rotation was decelerated above the perceptual threshold from 25°/s to 5°/s in 2 s and the helicopter emerged from the clouds. This is perceived as an acceleration, which conflicted with the turn direction as shown on the visuals. In the NoSD condition, the simulator was instead accelerated from −2°/s to 18°/s at *t* = 90 s, which matched the direction of the shown turn (see also Landman et al., 2024). After *t* = 120 s, the yaw rotation was faded out and the helicopter returned to straight and level flight.

#### Night Vision Goggles (NVGs)

The participant was in the monitoring role so that the operationally relevant task could be given to scan outside. The TEDAC was turned off to make this task more relevant and decrease light in the cockpit. The helicopter was flying near a coastline with lights, at 1100 ft altitude, and turned 90° to face the ocean at *t* = 90 s. In the SD condition, overall lumination was decreased and brightness of the stars and moon were set to 0% causing a complete loss of visual reference of the horizon. After *t* = 120 s, the helicopter turned back to show the coastline again.

### Auditory Cognitive Task

To simulate communication and navigation tasks, a mental tracking-type task was used, as this requires storage information in working memory as well as processing this information. A mental arithmetic task was selected (see Englund et al., 1987). Addition and subtraction problems were presented via the headset and consisted of two semi-randomly generated numbers between 1–9, separated by a randomly generated “plus” or “minus” operator. Additions never started with a number above 4, and subtractions never with a number below 6. Problems with the solution 5 were excluded. No same problems were presented in a row. Participants indicated whether the answer was above or below 5 using the Pilot Night Vision System (PNVS) switch on the collective. Ten problems were presented at a fixed pace in each run, before (*t* = 30–60 s), during (*t* = 90–120 s), and after the SD stimulus (*t* = 150–180 s).

### Dependent Measures

#### Auditory Cognitive Task Reaction Time (RT)

The main measure of auditory cognitive task performance is RT, defined as the time from which the audio of the last number of the problem started until the response via the PNVS switch. Answers outside of the available 1.5 s for answering were excluded and registered as error. The mean of the ten problems presented during the SD or NoSD stimulus (*t* = 90–120 s) was obtained. To correct for scenario context effects, the mean of the cognitive task sessions before and after stimulus presentation was then subtracted from this to obtain the ∆ RT.

#### Auditory Cognitive Task Accuracy

Auditory cognitive task accuracy was a secondary performance measure as a low number of useful data for statistical analysis were expected. It was defined as the proportion of correctly answered problems in each cognitive task session. Similar to RT, accuracy was obtained during stimulus presentation, and corrected by subtracting the mean of the sessions before and after stimulus presentation, to obtain the ∆ accuracy.

#### % Dwell Time on Attitude Indicator

One area of interest was defined, containing the right MPD (see Figure 3) which showed the flight page with the attitude indicator, speed, altitude, and heading. The percentage of dwell time within this area of interest with regard to the total dwell time during stimulus presentation was obtained. Invalid samples due to blinks were excluded from total dwell time. The flight page was not shown during the somatogyral yaw stimulus; therefore, this stimulus was excluded from analysis.

#### Flying Performance

As a manipulation check of SD, control inputs were registered in the actively flown scenarios (i.e., false horizon, featureless terrain, and leans). The root mean square (RMS) of the roll angle deviation from 0°, was obtained in the false horizon and leans scenario, whereas the RMS of the altitude deviation from the instructed altitude was obtained for the featureless terrain scenario, corresponding with the axis of the induced SD conflicts. A higher RMS means that pilots had higher deviations from the instructed flight state.

#### Subjective Measures

During debriefing, participants were explained each scenario and rated the severity of experienced SD and severity of experienced impact on cognitive performance of both conditions on a 1–5 Likert-type scale (labels: “Not at all,” “Weak,” “Moderate,” “Severe,” and “Very severe”). After each run, participants were asked whether they had noticed anything surprising or out of the ordinary. The percentage of participants who reported having been surprised by (effects of) the SD stimulus the first time they encountered it, was used to gauge stimuli awareness. In addition, to monitor motion sickness symptoms, participants rated the severity of motion sickness symptoms half-way into the test session and at the end on the Dutch version of the Misery Scale (MISC) ranging from 0 (“no problems”) to 10 (“vomiting”; Bos et al., 2005).

### Data Analysis

Alpha was set to 0.05 for the reporting of statistically significant results. Effect sizes of *d* = 0.2, 0.5, 0.8 and correspondingly *r* = 0.1, 0.3, 0.5, will be interpreted as small, medium, and large, respectively (Cohen, 2016). Cognitive task performance, gaze behavior, and self-reports of SD were compared between the SD and NoSD condition using a paired-samples *t* test, or with a Wilcoxon Signed Rank test if not normally distributed as determined using a Shapiro–Wilk test. This was done for the mean of all stimuli (main hypothesis) and for each stimulus separately (explorative).

### Data Loss

Simulator logging errors caused loss of performance data for the somatogyral SD stimulus of one participant in the second session. These performance data were imputed using the data for the somatogyral SD stimulus of the participant’s first session. A technical problem caused the loss of data of one other participant, but subjective measures were still obtained for all scenarios but the featureless terrain and brownout. Logging errors caused additional loss of performance for 80 arithmetic problems (1.7% of total data), divided over all participants. Of the eye-tracking data, 6.8% of samples did not contain gaze direction data, which could be caused by either blinks or measurement errors.

## Results

### Auditory Cognitive Task Performance

**Figure 5.**
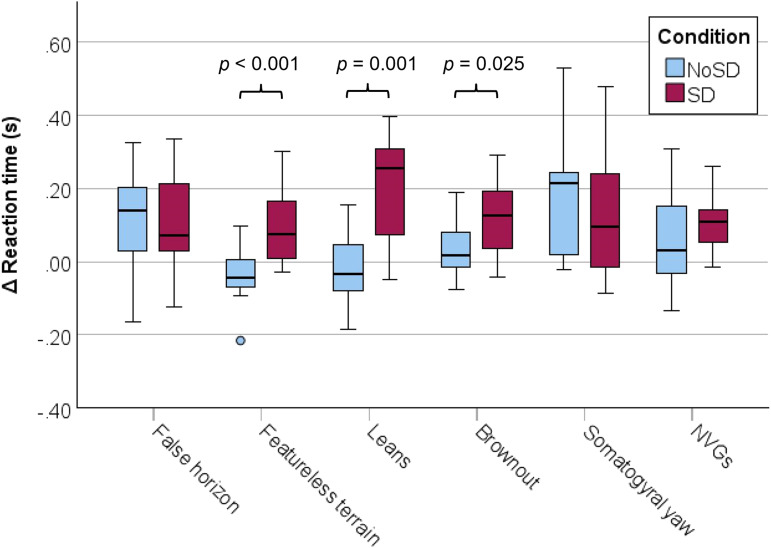
Tukey boxplots of the ∆ reaction time (stimulus – baseline) on the auditory cognitive task.

The overall ∆ RT (see Figure 5) was significantly higher in the SD condition than in the NoSD condition (Table 2). The effect size was large, *d* = 1.56. Reaction time during stimulus presentation increased by 15.4% compared to baseline in the SD condition and 7.1% in the NoSD condition. T-tests for specific stimuli showed that the ∆ RT was significantly higher in the SD condition for the featureless terrain, leans, and brownout stimuli, while there was no significant difference for the other stimuli.

**Table 2.** Results of SD-NoSD Comparisons of ∆ RT. Sdev = standard deviation.

	Mean ∆ RT (s), SDev		*t* (12)	*r*	*p*
	SD	NoSD			
Overall**	0.13, 0.06	0.05, 0.06	5.63	0.705	<0.001
False horizon	0.11, 0.13	0.11, 0.14	0.06	0.030	0.956
Featureless terrain**	0.09, 0.11	−0.04, 0.07	4.33	0.248	<0.001
Leans**	0.21, 0.14	−0.02, 0.09	4.11	−0.387	0.001
Brownout*	0.13, 0.10	0.04, 0.08	2.57	0.132	0.025
Somatogyral yaw	0.13, 0.16	0.18, 0.15	−1.79	0.777	0.099
NVGs	0.11, 0.08	0.05, 0.14	1.40	0.304	0.188

**p < 0.01, *p < 0.05.

The overall ∆ accuracy was significantly lower in the SD than in the NoSD condition, *Z* = −3.18, *p* = 0.033, with a large effect size, *r* = 0.602. Looking at specific stimuli, the ∆ accuracy was significantly lower in the SD condition only for the leans stimulus, *Z* = −2.76, *p* = 0.010, while there was no significant difference for the other stimuli.

### Gaze Behavior

**Figure 6.**
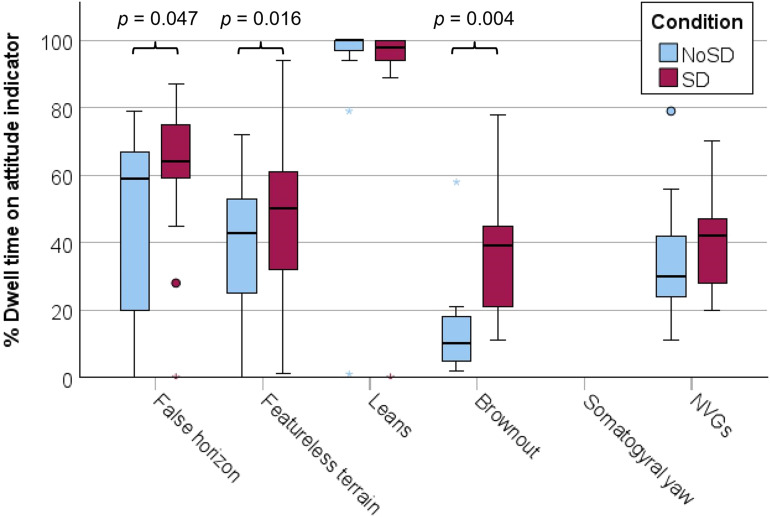
Tukey boxplots of the % dwell time on the attitude indicator. °: >2 Interquartile range, *: >3 Interquartile range.

The % gaze dwell time on the attitude indicator (see Figure 6), averaged for all stimuli, was significantly higher in the SD condition than in the NoSD condition (Table 3). The effect size was large, *d* = 1.37. For specific stimuli, the same effect was found to be significant for the false horizon, featureless terrain, and brownout stimuli, whereas there was no significant difference for the other stimuli (Table 3).

**Table 3.** Results of SD-NoSD Comparisons of Proportion of Dwell Time on the Attitude Indicator.

	Mean Proportion (%), SDev		*t* (12)	*r*	*p*
	SD	NoSD			
Overall**	55.7, 14.9	45.8, 16.8	4.92	0.904	<0.001
False horizon*	59.9, 23.7	50.4, 26.5	2.21	0.813	0.047
Featureless terrain*	48.4, 26.7	40.1, 23.2	2.82	0.919	0.016
Leans	89.8, 27.2	89.9, 27.3	0.14	0.990	0.888
Brownout**	38.1, 21.8	13.5, 14.9	3.59	0.145	0.004
Somatogyral yaw	N/A	N/A	N/A	N/A	N/A
NVGs	42.2, 15.6	35.2, 19.1	1.22	0.288	0.246

**p < 0.01, *p < 0.05.

### Subjective Measures

The subjective level of SD was significantly higher in the SD than in the NoSD conditions in general, and for all stimuli separately (Table 4). The subjectively experienced impact of the stimuli on cognitive performance was significantly higher in the SD condition, median = 2.9, IQR = 0.63, than in the NoSD condition, median = 1.8, IQR = 0.75, *Z* = −3.19, *p* = 0.001 (Table 5). This was also the case for all stimuli analyzed separately, except for the featureless terrain stimulus. For the false horizon SD stimulus, 54% of participants indicated recognition on the first exposure, 23% for featureless terrain, 100% for leans, 100% for brownout, 23% for somatogyral yaw, and 15% for NVGs. Self-reported motion sickness symptoms were on average very low halfway into the test session, M = 1.4, SDev = 1.9, as well as at the end M = 1.7, SDev = 1.9.

**Table 4.** Results of the SD-NoSD Comparisons of Subjective Level of SD.

	Median Rating (1–5), IQR		*Z* (12*)*	*p*
	SD	NoSD		
Overall**	3.3, 0.5	1.8, 0.5	−3.19	0.001
False horizon*	3.0, 1.0	2.0, 1.5	−2.52	0.012
Featureless terrain*	2.0, 0.5	2.0, 1.0	−2.24	0.025
Leans**	4.0, 1.5	2,0, 1.5	−3.25	0.001
Brownout**	4.0, 1.5	2.0, 1.0	−2.91	0.004
Somatogyral yaw**	4.0, 1.0	2.0, 1.0	−3.11	0.002
NVGs*	2.0, 1.0	2.0, 1.5	−2.45	0.014

IQR = interquartile range. **p < 0.01, *p < 0.05.

**Table 5.** Results of the SD-NoSD Comparisons of Subjective Impact of SD on Cognitive Performance.

	Median Rating (1–5), IQR		*Z* (12)	*p*
	SD	NoSD		
Overall	3.0, 0.4	1.8, 0.8	−3.19	0.001
False horizon**	3.0, 1.0	2.0, 1.0	−2.92	0.004
Featureless terrain	2.0, 1.5	2.0, 1.0	−1.73	0.083
Leans**	4,0, 1.25	2.0, 1.0	−3.10	0.002
Brownout**	3.0, 1.5	2.0, 1.5	−2.95	0.003
Somatogyral yaw*	3.0, 2,0	2.0, 0.5	−2.41	0.016
NVGs*	2.0, 1.5	2.0, 1.0	−2.00	0.046

IQR = interquartile range. **p < 0.01, *p < 0.05.

### Flying Performance

For the leans stimulus, the roll angle RMS was significantly higher in the SD condition, median = 3.08° than in the NoSD condition, median = 2.03° *Z* = −2.83, *p* = 0.005. There was no significant difference for the featureless terrain, *p* = 0.437, and false horizon, *p* = 0.116, stimuli.

## Discussion

The main objective of the study was to quantify the effect of SD stimuli on mental workload and attentional focus in military helicopter pilots. Six SD stimuli were used based on reported high prevalence in military aviation (Pennings et al., 2020). There was a significant, large negative effect of SD stimuli compared to NoSD stimuli on auditory cognitive task reaction time and accuracy, and pilots spent significantly more time looking at the artificial horizon in the SD condition. Self-reports by the pilots confirm these results, as both level of SD and the impact severity on cognition were rated significantly higher in the SD than in the NoSD condition, and none of the pilots reported having difficulty recalling the stimuli conditions in order to make their retrospective ratings. Our findings are in line with the “orientation first” principle (Gresty et al., 2003), which states that attentional focus is being allocated to self-orientation when spatial disorientation occurs. Our findings are also in line with previous simulator and lab studies (Landman et al., 2024; Strozak et al., 2018).

The secondary objective was to use these objective measures to explore which SD stimuli are (most) effective. A significant impact of SD stimuli on response time was found for the featureless terrain, leans, and brownout stimuli, but not for the false horizon, somatogyral and NVGs stimuli. For gaze behavior, a significant effect was found for the false horizon, featureless terrain, and brownout. The leans featured no outside visuals so that participants were looking mostly to the artificial horizon in both conditions. For this stimulus, other additional evidence of the impact of SD was found, as response accuracy was significantly lower and bank angle variability was significantly higher. However, the practical significance of the bank angle variability is questionable, as the difference between the SD and NoSD stimuli was only 1°. The somatogyral yaw and NVGs stimuli appeared to be ineffective at inducing SD.

The self-reports indicated that all stimuli, except the featureless terrain, were noticed by the pilots. Interestingly, the featureless terrain did induce significant effects on performance and gaze behavior, suggesting that SD may impact workload and attention without being recognized. The absence of significant auditory cognitive task performance effects for the false horizon contrasts with (nearly significant) findings by Strozak et al. (2018), who covered the attitude indicator for some time and forced pilots to orient themselves to the false horizon. The absence of significant effects in the somatogyral yaw scenario seems to result from an impact of the stimulus in both conditions. Pilots indicated that the unannounced turn, which occurred in both conditions, surprised them. The absence of significant effects in the NVGs scenario can be explained by the flight path being relatively steady; that is, pilots could easily regain orientation using the instruments. Only 15% of pilots indicated recognition of the NVGs SD stimulus. Both the somatogyral and NVGs scenarios featured a monitoring instead of an active flying task, which may have reduced effects of the SD stimuli.

The following limitations are important when considering the results. First, vestibular SD stimuli produced by ground-based simulators are different from the actual SD stimuli in flight, which may have affected results of the leans and somatogyral stimuli. Second, pilots were not flying their own aircraft type, meaning that performing the flying tasks, especially in instrument meteorological conditions (IMC), already induced high cognitive workload. Third, the decision to not inform pilots beforehand that they would be presented with SD stimuli may have caused undesired interpretations of events and tasks in some scenarios. For example, the brownout SD stimulus involves a hazardous situation which should normally be avoided by commanding a go-around, but this was not possible. The leans involved an unexpected vestibular motion cue, which could also be interpreted as an unexpected helicopter motion due to a mechanical failure.

In summary, better framing of the experimental tasks and stimuli would help manage expectations and prevent undesired interpretations of cues, but this would also limit the application of these stimuli in operational tasks to be used for research or scenario-based training. For research purposes, the scenarios should be kept more abstract, as this would also allow for the unrealistic momentarily covering of the instruments to induce SD. It would be interesting to use a more operationally relevant cognitive task in future research so that cognitive performance can be more directly related to operational performance. For ground-based training purposes, visual SD stimuli seem preferable, as these can be more integrated with realistic cues in scenarios where pilots remain in-the-loop.

In conclusion, we found empirical evidence of the “orientation first” principle in an ecologically relevant environment in a sample of military pilots. Using objective measures of cognitive performance and attentional focus, evidence of the impact of SD stimuli on pilot cognitive state was found. Practical applications of these findings include the use of these objective measures for checking scenario effectiveness for SD training and research purposes. The results also show that SD may reduce the mission effectiveness of military pilots who must process a large amount of tactical information.

## Key Points

• We demonstrate the relevance of two objective measures of the impact of spatially disorienting (SD) stimuli on pilot cognition: pilot secondary task performance and gaze behavior.
• Going beyond pilot control inputs, we show that these measures can be used to quantify the impact of SD stimuli on pilots’ mental state.
• Some tested SD stimuli induced large significant effects, whereas other stimuli induced no significant effects.
• Novel simulation technology was used, which integrated a six degrees-of-freedom motion-base platform with Virtual Reality presentation of visuals.
